# Elevated Erythritol: A Marker of Metabolic Dysregulation or Contributor to the Pathogenesis of Cardiometabolic Disease?

**DOI:** 10.3390/nu15184011

**Published:** 2023-09-16

**Authors:** Tagreed A. Mazi, Kimber L. Stanhope

**Affiliations:** 1 Department of Community Health Sciences-Clinical Nutrition, College of Applied Medical Sciences, King Saud University, P.O. Box 10219, Riyadh 11433, Saudi Arabia; 2 Department of Molecular Biosciences, School of Veterinary Medicine, University of California, Davis, CA 95616, USA; klstanhope@ucdavis.edu

**Keywords:** erythritol, cardiovascular disease, platelet activation, thrombosis, obesity, diabetes

## Abstract

Erythritol is a non-nutritive sugar replacement that can be endogenously produced by humans. Witkowski et al. reported that elevated circulating erythritol is associated with adverse cardiovascular events in three independent cohorts, demonstrated in vitro and ex vivo that erythritol promotes platelet activation, and showed faster clotting time in mice injected with erythritol. It was concluded that erythritol fosters enhanced thrombosis. This narrative review presents additional evidence that needs to be considered when evaluating these data and conclusions. We conducted a search of all studies related to erythritol exposure with focus on those that reported vascular health outcomes. Patients with chronically elevated erythritol levels due to inborn errors of metabolism do not exhibit higher platelet activation or thrombosis risk. Most long-term studies in which animals consumed high levels of erythritol do not support its role in platelet activation and thrombosis formation. Clinical data on the effects of chronic intake of erythritol are limited. Erythritol may be merely a marker of dysregulation in the Pentose Phosphate Pathway caused by impaired glycemia. However, this suggestion and the findings of Witkowski et al. need to be further examined. Clinical trials examining the long-term effects of erythritol consumption on cardiometabolic outcomes are required to test the causality between dietary erythritol and cardiometabolic risk. Until supportive data from these trials are available, it cannot be concluded that dietary erythritol promotes platelet activation, thrombosis, and cardiometabolic risk.

## 1. Introduction

Erythritol (1,2,3,4-butanetetrol) is a 4-carbon sugar alcohol that is found in many fruits and fermented foods and produced on a commercial scale by bacterial fermentation [1]. It is increasingly used as a sugar alternative, with adult dietary exposure expected to reach 30 gm/day [2]. As a replacement for sugar, erythritol has advantages over the other commonly consumed sugar alcohols: xylitol, sorbitol, mannitol and maltitol. It is better absorbed from the small intestine and, therefore, induces less undesirable gastrointestinal effects [3]. It contains negligible energy, while the other sugar alcohols contain 2–3 Kcal/gm [4]. It has also been shown that erythritol maintains and improves dental health more effectively than xylitol or sorbitol [5].

In healthy lean subjects, minimal amounts of a dietary erythritol dose are metabolized to erythronate (<1%), with the majority being unmetabolized and excreted in the urine unchanged [6]. In humans, erythritol is produced endogenously via erythrose-4-phosphate, an intermediate of the non-oxidative branch of the Pentose Phosphate Pathway (PPP). Erythrose-4-phosphate is the precursor of erythrose, which is converted to erythritol via NADPH-dependent reactions that are catalyzed by alcohol dehydrogenase 1 (encoded by *ADH1*) and sorbitol dehydrogenase (encoded by *SORD*) [7,8]. 

Pre-clinical evidence and short-term clinical studies have shown that erythritol may exert beneficial metabolic effects on body weight and glucose metabolism (reviewed in [3]). In contrast, prospective cohort studies indicate that elevated circulating erythritol levels are associated with a risk of adverse cardiometabolic outcomes, including central adiposity, diabetes, and cardiovascular disease (CVD) [7,9,10,11,12,13,14,15,16,17]. In a previous review, we suggested that erythritol may be a benign marker of PPP dysregulation caused by impaired glycemia (as in insulin resistance and diabetes) and/or by high-sugar diets [3]. A recent study, however, suggests a mechanism that may explain the association reported between erythritol and CVD. Witkowski et al. presented evidence from observational, in vitro, ex vivo, and in vivo studies demonstrating that erythritol promotes platelet activation and clot formation and concluded that erythritol fosters enhanced thrombosis [12]. Importantly, the authors did not present data demonstrating that consumption of erythritol promotes platelet activation and clot formation. In addition to noting this concern, in this review, we examine additional evidence that needs to be considered when evaluating the data and conclusions presented by Witkowski et al.

## 2. Erythritol Exposure Due to Inborn Errors of Pentose Phosphate Metabolism

Witkowski et al. [12] performed metabolomics profiling on 3 independent cohorts of patients undergoing elective cardiac risk assessment. Within 3 years of follow-up, elevated erythritol levels were found to be associated with risk of major adverse cardiovascular events (defined as death, non-fatal myocardial infarction, and non-fatal stroke). This finding, and similar findings from other observational studies [7,9,10,11,12,13,14,15,16,17], do not establish causality between elevated circulating erythritol and increased risk of cardiometabolic disease. Furthermore, none of these studies assessed dietary intake of erythritol in order to demonstrate an association between consumption of erythritol and cardiometabolic risk. Indeed, in some of these studies, the blood in which erythritol levels were assessed was collected before erythritol was approved as a food additive in the U.S. [10,13,18]. In others [7,9,15,16], the global per capita estimates for erythritol consumption (e.g., 0.023 g/day in 2019) seem too low to implicate dietary erythritol for a role in the positive associations reported between circulating erythritol and cardiometabolic disease [19,20]. However, Witkowski et al. showed that circulating erythritol increased over 1000-fold for 24 h after 8 study participants consumed a commercial food product containing 30 g of erythritol. This suggests that if high levels of circulating erythritol promote platelet activation and clot formation, then consumption of erythritol does, too. 

Therefore, it is of interest to further explore the link between high levels of circulating erythritol and platelet activation and clot formation by examining conditions in which circulating erythritol is chronically elevated due to other factors, such as genetic metabolic disorders. In two inborn errors of metabolism caused by the genetic deficiencies of transketolase (encoded by *TKT*) or transaldolase (encoded by *TALDO1*), key enzymes in the non-oxidative branch of the PPP, chronic elevations of endogenously produced erythritol, and other polyols, are observed in both plasma and urine [21]. Clinical signs and symptoms typically manifest in the neonatal period and progress throughout life. In both conditions, obesity, diabetes, or other cardiometabolic dysregulations are not reported, and cardiac conditions observed are related to congenital birth defects [21,22,23,24,25,26,27,28,29,30]. In transaldolase deficiency, congenital coagulopathies (blood clotting disorders) are commonly reported. However, rather than upregulated platelet activation and hypercoagulation, patients display thrombocytopenia with abnormalities including prolonged prothrombin, thrombin, and activated partial thromboplastin times [22,23,24,25,26,27,28,29,31,32,33,34,35]. In other words, these patients display bleeding tendencies rather than tendencies toward clot formation. Other symptoms include hepatosplenomegaly, which may progress to liver cirrhosis and dysfunction [21]. While the coagulopathies reported could be attributed to concurrent liver dysfunction [36], there are multiple reports of cases with neonatal bleeding tendency, thrombocytopenia, and abnormal coagulation profile that were observed prior to abnormal liver function [25,26,37]. In transketolase deficiency, hepatic abnormalities are rare, and neither hyper- nor hypo-coagulation were reported [21,30]. Another genetic inborn error of PPP, sedoheptulo kinase deficiency (encoded by SHPK), is also characterized by increased endogenous erythritol synthesis [38] and increased urinary erythritol [38,39]. The available case report described two patients, and coagulation abnormalities were not reported [38]. Whereas the generalizability of these case reports from patients with inborn errors of PPP may be limited, they do not indicate that chronically elevated circulating erythritol promotes hypercoagulation or the development of thrombosis.

## 3. Erythritol Exposure: Effects on Vascular Health from In Vitro Studies

In ex vivo and in vitro studies, Witkowski et al. [12] demonstrated erythritol’s potential in augmenting platelet activation and aggregation response. In human platelet-rich plasma (from healthy volunteers), the exposure to erythritol (45–270 μM) versus normal saline resulted in a dose-dependent increase in agonist-stimulated platelet aggregation response. Using washed human platelets, the authors showed that erythritol treatment, compared to vehicle-enhanced agonists-induced cytosolic calcium levels, caused a dose-dependent increase in P-selectin surface expression and activated glycoprotein α2β3 (GP IIb/IIIa), which indicate platelet activation. Additionally, in human whole blood, erythritol exposure augmented the rate of collagen-dependent platelet adhesion and thrombus formation. These findings are in line with other in vitro studies which indicate that erythritol exposure may adversely affect outcomes related to vascular health. In one study, endothelial progenitor cells harvested from male mice after 6 weeks of oral erythritol intake (15 mg/kg) showed impaired function (measured by migration, tube formation, and adhesion) compared with cells harvested from mice that consumed water [40]. It was concluded that erythritol exposure results in the dysfunction of endothelial progenitor cells and may impair local angiogenesis and potentially exacerbate thrombotic events [40]. In human leukemia monocytes (THP-1-derived macrophages), treatment with erythritol (1 mM) compared to vehicle resulted in the upregulation of markers of the pro-inflammatory M1-like macrophages, increased levels of reactive oxygen species and cytosolic calcium, activated the AKT signaling pathway and upregulated cell cycle arrest via necrosis [41]. The authors concluded that erythritol may induce inflammation and necroptosis [41]. 

In contrast to these findings, other in vitro studies showed that erythritol exposure may promote favorable effects on vascular health. In one study, when human umbilical vein endothelial cells were treated with normal and high glucose (7 or 30 mM) and exposed to diabetic stressors, erythritol treatment protected against cell death and lowered nitric oxide release in the high-glucose cells, with no effect observed in the normal-glucose group [42]. In addition, the same study showed that erythritol treatment reversed the alterations in gene transcripts linked to inflammation and endothelial dysfunction, and the authors concluded that erythritol induced endothelial protective effects under hyperglycemic conditions [42]. In another study conducted in red blood cells, erythritol treatment (0–50 mM) dose-dependently prevented free radical-induced bio-membrane oxidative damage and hemolysis, suggesting that erythritol has antioxidant potential [43]. It is possible that the discrepancy in the in vitro studies findings relates to the types of cells or biological tissues studied as well as to the different comparator compounds used.

## 4. Erythritol Exposure: Injection vs. Oral Intake

Using a mouse model of arterial injury, Witkowski et al. [12] examined the effects of acute exposure to erythritol by direct injection into the carotid vein (25 mg kg^−1^) on in vivo thrombosis parameters. Compared to injection of normal saline or 1,5-anhydroglucitol, erythritol caused a higher rate of clot formation and a reduction in time to cessation of blood flow after arterial injury. While these findings support a role of erythritol in thrombosis, it is important to note that multiple stimuli can provoke platelet activation besides vessel wall injury, including changes in osmotic pressure. Erythritol has a molecular weight of 122.12 gm/mol, which is lower than the molecular weight of the other sugar alcohols, 1,5-anhydroglucitol, or glucose. Therefore, on a per-gram basis, it will exert more osmotic pressure than the other sugar alcohols or glucose. A large oral bolus dose of erythritol (1 gm/kg in 250-mL solution) is reported to induce osmotic gastrointestinal effects [44]. In general, hyperosmolar solutions that are administered intravenously result in a shift of fluids from the intracellular to the extracellular compartment and a subsequent increase in blood volume and pressure. This response is affected by concentration, number of solute particles, volume, and rate of injection. In response to these changes, platelets, along with other blood cells, may become activated. In support of this, the intravenous infusion of the sugar alcohol mannitol (molecular weight of 182.17 gm/mol) in rabbit ears, as compared to normal saline (isotonic solution), increased levels of tissue factor and von Willebrand factor, indicating thrombus formation, with the effect increasing with infusion time [45]. The authors concluded that intravenously administered mannitol may induce hyperosmotic effects, resulting in the dehydration of vascular endothelial cells, local platelet response, and coagulation. In mice, the renal cortical injection of mannitol (200 mg) resulted in increased blood flow, suggesting a hyperosmotic effect, and thrombosis was detected in glomerular capillaries 35 min post-injection [46]. Therefore, the intravenous injection of erythritol that was administered in the study by Witkowski et al. [12], which does not represent the physiological conditions caused by oral consumption, could have induced a hyperosmotic effect that was possibly responsible for the platelet activation and coagulation response.

There are, however, studies that examined the effects of chronic oral intake of erythritol on parameters related to coagulation, and the results are inconsistent. In one study, using a cerebral ischemia animal model, male mice were provided with erythritol (15 mg/kg) in drinking water for 6 weeks and then subjected to electrocoagulation of the middle cerebral artery [40]. Compared to control (water), erythritol resulted in lower capillary density and an increase in thrombosis infarct volume, which suggests that long-term erythritol consumption aggravates cerebral ischemia [40]. However, these findings are not in agreement with other studies. In rats consuming diets containing 0 (control), 5, or 10% erythritol or 10% mannitol for 104–107 weeks, no changes were observed in prothrombin time, thrombocytes count, platelets volume and platelets distribution width measured at week 26, 51, 78, and 103 [47]. Of note, the 10% erythritol dose is equivalent to approximately 5 gm/kg/day, which is five times higher than the usual highest dose administered to human subjects. In another study conducted in male rats, feeding diets containing erythritol at 0 (control), 5, or 10% for 4 weeks caused no significant differences between treatment groups versus controls in thrombocyte count, prothrombin and activated partial thromboplastin times, mean platelet volume, and platelet distribution width [45]. However, in female rats, 5% erythritol resulted in decreased thrombocyte count, and 10% erythritol resulted in prolonged prothrombin time [48], which indicates less blood clotting potential. In male rats, dietary erythritol at levels of 0 (control), 5, 10, or 20% over 12 weeks resulted in prolonged prothrombin time in the 20% group compared to control, with no changes observed in other coagulation outcomes [49]. In a study conducted in mice, erythritol incorporated in diets at levels of 0 (control), 5, 10, or 20% for 12 weeks showed no effects on thrombocyte counts, prothrombin time, mean platelet volume, or platelet distribution width [49]. In a study conducted in dogs, 53 weeks of erythritol feeding at levels of 0 (controls), 2, 5, or 10% showed no effect on prothrombin time [50]. While the authors reported spontaneous abnormal changes in platelet count values throughout the study, these data were not shown, and the direction of change was not specified. Together, the majority of studies that tested long-term consumption of diets containing high levels of erythritol (4 to 104 weeks) did not demonstrate potential for platelet activation or enhanced thrombosis and do not corroborate the acute in vivo clot-forming effects of injected erythritol as reported by Witkowski et al. [12].

## 5. Erythritol Exposure: Effects on Vascular Health in High-Risk Groups

The findings by Witkowski et al. [12] suggest that erythritol may increase thrombotic potentials in high-risk groups in which obesity and other cardiometabolic risk factors are prevalent. This, as noted by the authors, is especially concerning as sugar replacements, including erythritol, are often recommended for these populations. However, as already stated, results from Witkowski et al. do not relate the effects of dietary consumption of erythritol to thrombotic processes. Currently, clinical data on the long-term effects of dietary erythritol on cardiometabolic outcomes in high-risk subjects are almost completely lacking, with only one human study available. Flint et al. examined the effect of dietary consumption of erythritol (36 gm/day) for 4 weeks on vascular health in subjects with type 2 diabetes. Results showed that erythritol reduced arterial stiffness (when measured by central pulse pressure) and improved fingertip endothelial function. In subjects with systolic blood pressure greater than 130 mm/hg, erythritol consumption lowered systolic blood pressure [51]. However, erythritol had no effects on inflammation (as measured by C-reactive protein) and oxidative stress (as measured by urinary prostaglandin F2α) [51]. As noted by the authors, the study is limited by the lack of a control group and lack of blinding. 

Results from studies in rodents with streptozotocin-induced diabetes or with obesity are in line with the above results. In diabetic compared with normoglycemic rats, 3 weeks of erythritol administration (1 gm/kg) in drinking water preserved endothelium-dependent relaxation function (determined ex vivo by measuring the contraction response of isolated aortic rings to stimulants). No effects were observed on antioxidant capacity (as measured by Trolox Equivalent Antioxidant Capacity and circulating reduced and oxidized glutathione) [43]. In another study, male diabetic rats were fed fructose for 2 weeks to induce insulin resistance and then provided 0, 5, 10, or 20% erythritol in drinking water for 8 weeks. Compared to 0%, consumption of erythritol resulted in less insulin resistance and lowered circulating cholesterol, LDL-cholesterol, and triglycerides concentrations. Erythritol also improved serum markers of lipid peroxidation (as measured by malondialdehyde) and antioxidant capacity/oxidative stress (as measured by glutathione and superoxide dismutase) compared to 0%. However, within 2 weeks of diabetes induction and 1 week of diet treatment, two animals in the 10% group and four animals in the 20% group experienced hypoglycemia and mortality [52]. In mice with obesity due to a high-fat diet, the addition of 5% erythritol to the diet improved glucose tolerance and lowered body weight, adiposity, serum lipids, hepatocellular lipid accumulation, and inflammatory gene expression compared to high-fat alone [53]. Together, these data suggest that chronic dietary intake of erythritol does not adversely affect cardiometabolic health in vulnerable populations and may have endothelium-protective effects; however, more studies, especially clinical dietary intervention trials, are needed. 

## 6. Erythritol Exposure: Heightened by Impaired Glycemia and High Sugar Diets?

Considering clinical reports of chronically elevated circulating erythritol in some inborn errors of PPP metabolism, the data from in vivo studies examining the effects of long-term dietary erythritol intake, and the inconsistencies in the results from in vitro studies investigating erythritol exposure, it is too early to conclude that platelet activation and enhanced thrombosis is the underlying mechanism explaining the association between circulating erythritol and CVD risk. It is possible, as we previously suggested [3], that erythritol is a benign marker of PPP dysregulation caused by impaired glycemia (hyperglycemia due to insulin resistance or diabetes) or high-sugar diets. Impaired glycemia [54,55,56] and high sugar diets [57] are risk factors for adverse cardiometabolic outcomes in diabetic and non-diabetic subjects.

Erythritol exerts no effects on insulin and blood glucose levels [51,58,59,60]; therefore, it does not induce hyperglycemia. However, there is evidence suggesting that hyperglycemia may increase erythritol levels from endogenous sources (discussed in [3]). In an in vitro study conducted in Human A549 lung carcinoma cells, erythritol levels increased dose-dependently with the increase in media glucose concentration, and cells cultured in high glucose (25 mM), which mimics hyperglycemia, had higher erythritol levels compared to low glucose (6.25 mM) [61]. It was concluded that endogenous erythritol synthesis is upregulated by increased substrate availability. In the same study, the chemical and genetic induction of oxidative stress also resulted in an increase in erythritol levels [61]. These findings indicate that increased glucose availability and oxidative stress, both of which characterize hyperglycemic states, may result in an upregulation of endogenous erythritol synthesis. Data from epidemiological studies also suggest that elevated erythritol is a parallel occurrence to the metabolic dysregulation associated with pre-diabetes, which includes hyperglycemia and insulin resistance. In one study, elevated erythritol was detected up to 20 years before diabetes diagnosis [10]. Another study that showed that plasma erythritol was a predictive biomarker for diabetes (OR (95%CI) 1.31 (1.02–1.69)) also reported that plasma glucose was a much stronger predictive biomarker (OR (95%CI) 5.5 (3.57–8.48)) [9].

Endogenous erythritol synthesis may also respond to diet-induced increases in glucose availability. In a recent study by Ortiz et al., the consumption of 30% sucrose in water for 2, 5, and 8 weeks elevated nonfasted plasma erythritol concentration in young male mice fed low-fat diets (4.5-fold at week 2) and high-fat diet (2.6-fold at week 2) compared with water controls [62]. It is interesting that consumption of sucrose with a low-fat diet led to higher circulating erythritol than sucrose with a high-fat diet because when the low-fat and high-fat diets were consumed without sucrose, there was no difference observed in erythritol concentrations. However, the authors noted that the sucrose group on the low-fat diet consumed 55% more sucrose water than the sucrose group on the high-fat diet and concluded that endogenous erythritol synthesis is a disposal pathway for glucose carbons when surplus sucrose is consumed [62]. Whether the fructose component in sucrose also has a role in promoting erythritol synthesis has not been studied.

Witkowski et al. [12] did not report the glucose concentrations of their observational cohorts nor an index of insulin resistance, but their data allow for the possibility that dysregulation of glucose metabolism could be involved in the increased erythritol levels. For all the three cohorts studied, as erythritol levels increased, the prevalence of diabetes increased, as well (*p* < 0.001) (Supplementary Tables S1–S3 in [12]). Additionally, including diabetes and the other covariates in the adjusted statistical models lowered the associations between erythritol and adverse cardiovascular events, and this observation was consistent in all three cohorts (Supplementary Tables S7–S9 in [12]). It would be interesting to know whether the associations would have been further attenuated with adjustment for a marker of insulin resistance such as the Homeostatic Model Assessment for Insulin Resistance. In the high-risk populations studied [12], it is likely the prevalence of insulin resistance was high even in the subjects who did not have diabetes.

**Figure 1:**
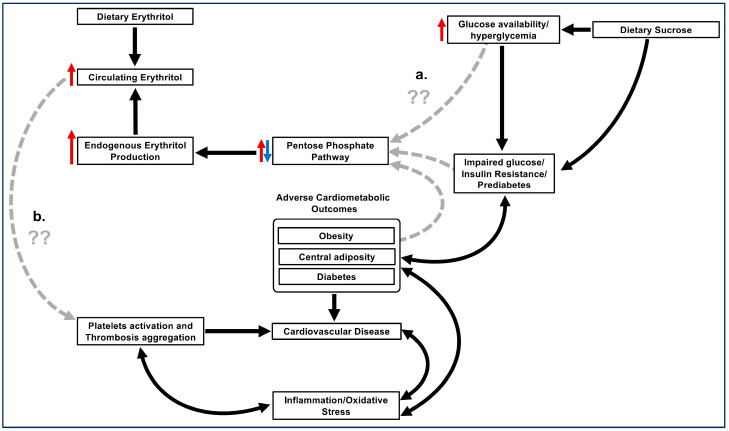
Hypothetical framework explaining the associations between circulating erythritol levels and adverse cardiometabolic outcomes. (**a**) Elevated circulating erythritol may result from a dysregulated Pentose Phosphate Pathway secondary to impaired glycemia (hyperglycemia, insulin resistance, and diabetes) or due to a high-sugar diet. Impaired glycemia is a risk factor for adverse cardiometabolic outcomes. (**b**) Elevated circulating erythritol may activate platelets and enhance aggregation. This may directly increase the risk of cardiovascular disease and indirectly increase the risk of other cardiometabolic diseases via increased inflammation and oxidative stress. The arrows designated in grey are hypothetical and require further research.

In summary, while epidemiological data [12] support the hypothesis that erythritol mediates an increased risk of CVD, it may also support the hypothesis that erythritol is merely a marker of impaired glucose/insulin resistance/diabetes and high sugar diets, which are well-established mediators of increased risk of CVD (Figure 1). Only long-term clinical dietary intervention studies will determine which, or if either, of these scenarios is accurate.

## 7. Conclusions

Prospective studies have shown that elevated circulating erythritol levels are associated with unfavorable cardiometabolic outcomes [7,9,10,11,12,13,14,15,16,17]. Witkowski et al. [12] suggest the association between elevated circulating erythritol levels and adverse cardiovascular outcomes is mediated by erythritol-promoting platelet activation and hypercoagulation. This mechanism, however, is not supported by clinical reports of inborn errors of PPP that do not link chronically elevated erythritol levels with higher thrombosis risk. The increased thrombosis risk reported following intravenous injection of erythritol into mice is not corroborated by the results from the majority of long-term animal studies, which demonstrate that chronic consumption of erythritol does not affect parameters related to coagulation. Establishing causality in the relationships observed in epidemiological studies requires clinical trials that examine the long-term effect of erythritol consumption on outcomes related to cardiometabolic health, especially in persons with obesity and diabetes. Until these data are available, it cannot be concluded that dietary erythritol promotes platelet activation and cardiometabolic risk.
